# Numeric Databases for Chemical Analysis

**DOI:** 10.6028/jres.094.005

**Published:** 1989

**Authors:** Sharon G. Lias

**Affiliations:** National Institute of Standards and Technology Gaithersburg, MD 20899

**Keywords:** analytical chemistry, computer, database, evaluation, infrared spectrum, mass spectrum, nuclear magnetic resonance

## Abstract

Databases for use with analytical chemistry instrumental techniques are surveyed, with attention to existing databases and collection efforts now underway, as well as needs for new data-bases. Collections of spectra for use in NMR, infrared spectroscopy, and mass spectroscopy are described. Using mass spectral databases as an example, a critique is presented of automated quality control procedures used to evaluate individual spectra in large collections; the kinds of problems which have been en-countered in using these procedures are discussed. Finally, a brief critical review is presented covering the application of computers to the identification of unknown compounds using spectral data-bases; again, algorithms used with mass spectrometry are taken as the example. Ongoing work at NIST with the NIST/EPA/MSDC Mass Spectral Database is concerned with many of these problems; recent developments are described.

## 1. Introduction

In principle, the measurement technique in which spectroscopy is used as an analytical tool involves obtaining a spectrum of the sample of interest (the “unknown”) and identifying the unknown compound by the similarity of its spectrum to that of a particular (“known”) chemical compound. Here we use the word “spectroscopy” in the broadest possible sense; spectroscopy is taken to be any experimental technique which provides a reproducible “spectrum” characteristic of particular chemical species. This includes, for example, all optical spectroscopy, nuclear magnetic resonance, electron spin resonance, mass spectrometry, and so on.

Of course, from the beginning of the use of spectral techniques in the analytical laboratory, it was recognized that the comparison spectra need not be obtained at the same time, or even on the same instrument, as the analysis itself. Because one could collect standard spectra and use them over and over again, it is not unexpected to find that there is a long history of data collection efforts aimed at analytical applications [1,2]. With the beginning of the computer age, it was of course a natural extension of these activities to store spectral databases on computers, and to conduct automated searches of those databases in order to “match” the spectrum of the unknown compound with that of a standard reference compound. The use of automated instruments equipped with reference libraries has become a well-established measurement technique for analytical chemistry. At the present time, computerized algorithms are also used to evaluate the large numbers of spectra which comprise these collections.

In spite of the long history of data collection efforts involving analytical spectra, there is some dis-satisfaction with the size and quality of available collections. For example, in 1986 Thomas L. Isen-hour wrote an editorial [3] describing the consensus of experts concerning computerized databases for use in analytical chemistry measurement techniques: “… the current state of spectroscopic data-bases is such that it inhibits good applications of known search and interpretive procedures as well as further research on these methods. … we do not in general have high quality spectroscopic data-bases available… Perhaps 10 million chemical compounds are now known. Some measurements have been made on all of them. Very few, if any, structure identifications have been made in recent times without resorting to some form of spectroscopy. Why then are the largest available spectral data files in computer format limited to a few tens of thousands of compounds?”

This paper presents a brief survey of the use of automated databases as an integral part of spectroscopic measurement techniques for analytical chemistry, with emphasis on mass spectrometric databases. Because of the rather dim view by the experts of the analytical databases in common use, the survey includes a list of the most popular automated analytical databases with attention to the numbers of spectra available in each of them. A discussion of the current state of automated evaluation algorithms being used with mass spectral data-bases is included.

Ongoing work aimed at updating and improving the quality of the mass spectrometric database distributed by the National Institute of Standards and Technology Office of Standard Reference Data is described.

## 2. Brief Survey of Automated Analytical Databases

### 2.1 Nuclear Magnetic Resonance Spectroscopy (NMR)

The databases listed below are all provided with software which enables the user to look up particular spectra or to match the characteristics of a particular spectrum of an unknown compound. Most NMR databases also include software for spectrum estimation and interpretation.

#### 2.1.1 C-13 NMR Database on the Chemical Information System

The Chemical Information System [4] collection currently consists of a total of 11,700 ^13^C NMR spectra. The database was last updated in November 1985, when many incorrect assignments in older spectra were corrected, and over 4,000 new spectra were added. The database was originally put together by the Royal Dutch Chemical Society (also called Netherlands Information Combine).

#### 2.1.2 C-13 NMR Online Service of the Fachinformationszentrum (FIZ), Karlsruhe, W. Germany (accessed in the U.S. through STN International)

This widely-used NMR database was added to the STN system [5] in December 1987, having been marketed previously in the U.S. by Scientific Information Service (SIS). The collection contains 67,500 ^13^C chemical shifts, coupling constants, and relaxation times.

#### 2.1.3 Bruker Spectroscopic Database

This data-base is available to Bruker customers for on-site use. It requires a Bruker Aspect 2000 or 3000 computer together with a Bruker software package (BASIS—Bruker Automatic Spectroscopy Interpretation System). The database contains various modules, including ^13^C NMR (19,000 spectra), ^1^H NMR (900 spectra), as well as a combined ^13^NMR-MS database.

#### 2.1.4 Sadtler Laboratories

This database consists of 24,000 sets of ^13^C NMR chemical shifts with compound names, and also 10,000 ^13^C NMR spectra in full digital format that can be used to view expanded displays of the spectra [1c]. The database is designed for use with Sadtler’s own ^13^C search software package, which operates on IBM-compatible personal computers.

#### 2.1.5 Collection of National Chemical Laboratory for Industry, Japan

The integrated online “Spectrum Database System” [6], which includes collections of NMR, ESR, IR, Raman, and mass spectra has both ^1^H NMR spectra (6,000 compounds) and ^13^C NMR spectra (5,700 compounds) along with search software enabling a user to look up a particular spectrum (and conditions under which it was run) or to match an unknown spectrum. All spectra were determined at the NCLI under carefully controlled conditions.

#### 2.1.6 Other Collections of NMR Spectra

The list given above is not exhaustive. For example, Varian also markets an NMR database, and Tsukuba University (Japan) produces a CD-ROM collection of ^13^C NMR spectra of polymers. The data came from existing handbooks. The system also contains programs to synthesize the NMR spectra from structural information.

### 2.2 Infrared Spectra (IR)

In the field of infrared spectroscopy, many large collections of spectra were built up [1,7] at a time when the spectrometers in use were prism and grating instruments. Within the past decade, the instrumentation in general use in analytical laboratories has changed to Fourier transform infrared spectrometers (FT-IR), which generate digitized spectra. Although the older analogue spectra can be digitized to be made compatible with the data systems of the newer instruments, questions have been raised about the desirability of doing this. In the opinion of some experts [8], many of the older collections of spectra are no longer adequate to serve as reference spectra for comparison with results taken on the newer instruments. For this reason, effort has been given recently to building completely new collections of IR spectra which were generated in digital format in FT-IR instruments. In the discussion which follows, attempts will be made to distinguish between the newer digitized collections, and databases of spectra from prism and grating spectrometers.

#### 2.2.1 Aldrich-Nicolet Digital FT-IR Database and the Sigma-Nicolet Biochemical Library

Nicolet, in collaboration with Aldrich and with Sigma, is producing high quality databases of FT-IR spectra of the compounds in the catalogues of these two companies. The Aldrich-Nicolet collection contained 10,600 compounds and the Sigma-Nicolet collection, 10,400 compounds in 1987. These databases are being updated in 1988 with the addition of several thousand new spectra. The databases are designed for use on several popular personal computers, and are distributed with software which is geared to locating spectra which match the peak intensities and locations from an IR spectrum of an unknown substance.

#### 2.2.2 Sadtler Research Laboratories Spectra

The largest commercially available collection of infrared spectra [1c], with > 60,000 spectra largely from prism and grating spectrometers. The current collection also includes some FT-IR spectra.

#### 2.2.3 Coblentz Society Spectra

Beginning in the mid-1960s, the Coblentz Society, in collaboration with the Joint Committee on Atomic and Molecular Physical Data (JCAMP), put together a collection of 10,500 donated infrared spectra taken on prism and grating spectrometers. The effort included developing evaluation procedures for IR spectra, and evaluating the entire collection of spectra. The collection was originally distributed in 10 volumes in a looseleaf notebook format [7]. Recently, 4,400 of these spectra have been digitized, and will be made available through the Coblentz Society, which is also digitizing the remaining spectra. Dr. Clara Craver, of the Chemir Labs, who played a key role in putting together the original Coblentz Society collection, is actively soliciting donations of new spectra to increase the size of the database, which will be available in a format for use with personal computers.

#### 2.2.4 EPA Vapor Phase Spectra

This collection of 3,300 spectra originated in laboratories of the EPA, and is in the public domain. Although not commercially available as a collection, the spectra are available through the instrument companies manufacturing IR spectrometers.

#### 2.2.5 Collection of the Univeristy of California-Riverside “Clearinghouse for Digital Infrared Spectra”

A new project was initiated in October 1986 for the collection of a database of digitized FT-IR spectra under the leadership of Drs. Peter Griffiths and Charles Wilkins at the University of California-Riverside. They hope to tap several collections of high quality digital spectra measured in various analytical laboratories for internal use. This team has put together an automated algorithm for evaluating the spectra of this collection [8].

#### 2.2.6 Infrared Data Committee of Japan (IRDC)

This organization has distributed IR spectra in printed form on edge-punched cards since 1961 [Id]. About 19,000 cards are now available. In 1980–85, the peak wavenumbers and intensities were extracted and entered into a computer file. Search software for the database has been prepared. A search involves entering wavenumbers and intensities in order of decreasing intensity; no-band regions can be specified. Spectra which are retrieved in a search are listed in order of the probability of being a correct match. The publisher of the IRDC cards is also marketing the above system in magnetic tape form. The possibility of fully digitizing the IRDC spectra has been discussed, but no decisions have been made.

#### 2.2.7 Collection of National Chemical Laboratory for Industry, Japan

The integrated online “Spectrum Database System” [6], which includes collections of NMR, ESR, IR, Raman, and mass spectra, also makes available a database of 22,500 infrared spectra. All spectra were determined at the NCLI under carefully controlled conditions. Data were transferred in digital form directly from the FT-IR instrument on which they were determined to the database. The database is available online to users in Japan.

#### 2.2.8 American Society for Testing and Materials Collection

Comprehensive indices coded by ASTM Committee E-13.03 for the infrared spectra from most of the older general collections are available from Chemir Labs, Sadtler Research Labs, and on-line on the Canadian Scientific Numeric Data System. Data for 145,000 compounds are included.

#### 2.2.9 Other Collections

Many hard-copy collections of IR spectra exist. For a comprehensive list of the numerous older collections, the reader is referred to the bibliography given in *The Coblentz Society Desk Book of Infrared Spectra* [9]. Paragraphs 2.2.3 and 2.2.5 describe new collection efforts aimed at the production of computerized IR databases. In addition, there are apparently several similar efforts now being initiated in Europe, notably at the University of Essen [10].

### 2.3 Mass Spectra

#### 2.3.1 The Wiley Registry of Mass Spectral Data

This collection has been put together and is maintained by F. W. McLafferty at Cornell University. The database, available from John Wiley & Sons, Inc. on magnetic tape or in a CD-ROM version, contains 123,704 spectra of 108,173 compounds evaluated using a Quality Index algorithm [11] (see discussion below). Replicate spectra of a given compound are included. The magnetic tape version is distributed without search software, although software for matching unknown spectra which is tailored to this database is available free of charge from Cornell University [12–17].

#### 2.3.2 The NIST/EPA/MSDC Mass Spectral Database

This database was originally put together by Drs. S. R. Heller and G. W. A. Milne of EPA and NIH, and called the EPA/NIH Mass Spectral Database. Since 1978, this database has been jointly administered by NIST and EPA, and new spectra are identified in the published literature, collected in complete form from the original authors, and evaluated by the Mass Spectrometry Data Center (MSDC), Nottingham, England. The current database consists of 43,005 spectra, each one corresponding to a unique chemical compound. Spectra in the current version of the data-base were selected from an archive of 79,000 mass spectra and evaluated using a Quality Index algorithm, based on—but not exactly the same as—the algorithm developed by F. W. McLafferty to evaluate the Wiley database [18,19]. (The Quality Index evaluations are discussed in detail in sec. 4.) The database is distributed on tape without search software, and in a PC version with search software and elementary matching software. A new update, which will include several thousand new spectra, is being prepared for release in the fall of 1988. The corresponding PC-version will incorporate structural information on all compounds in the database, as well as several new modes of matching spectra of unknown compounds to spectra in the database.

#### 2.3.3 The Merged Wiley/NBS Registry of Mass Spectral Data

The Wiley and NBS/EPA/MSDC collections are also available from John Wiley & Sons in a merged version, which has a total of 130,544 spectra (number of duplicate spectra in the two databases, 36,847). The merged database is available on tape and CD-ROM. A book version of the Merged Database is being published [20].

#### 2.3.4 The Eight Peak Index

The primary publication of the Mass Spectrometry Data Center, (Royal Society of Chemistry, Nottingham, England) is made up of a set of seven volumes [21] including 65,000 eight-peak spectra of 52,332 compounds indexed by molecular weight, chemical formula, and most abundant ions. This collection of partial spectra is also available on tape. The collection includes many of the same spectra included in the Wiley and NBS/EPA/MSDC collections. All of these collections of mass spectra have been put together incorporating older (non-computerized) data collections such as the spectra from the API Project 44 [1a], the Thermodynamics Research Center [1b], and the American Society for Testing and Materials (ASTM) [1e].

#### 2.3.5 Collection of National Chemical Laboratory for Industry, Japan

The integrated online “Spectrum Database System” [6], which also includes collections of NMR, ESR, IR, and Raman spectra has a database of 10,000 mass spectra which were determined in the NCLI laboratories as part of the larger project. The system was recently made available to users in Japan.

#### 2.3.6 Other Collections

(a) Japan Information System for Science and Technology (JICST) has an online "Mass Spectral Database System" searchable by name, formula. Chemical Abstracts Registry Number, and peaks. This system uses the NIST/EPA/MSDC database augmented by a collection of 6,000 spectra from the Mass Spectrometry Society of Japan, (b) Dr. D. Henneberg (Max-Planck-Institut fur Kohlenforschung) has a collection of approximately 12,000 spectra, to which he is adding with the intention of building a database [22].

## 3. Methods of Building Spectral Collections

While the above hsts make it clear that many collections of spectra for use in analytical chemistry laboratories are available, it is also evident that Thomas Isenhour’s complaint [3] that none of the collections contain more than about 100,000 spectra is also substantially correct. In order to understand why the sizes of available collections are so small even after several decades of collection effort (even excluding infrared spectroscopy, where earlier collections became less useful with the advent of new instrumentation), it is of interest to examine the techniques which are commonly used to collect spectra for such databases. This discussion will also consider how the nature and quality of a database is influenced by the way in which it has been put together.

### 3.1 Laboratory Efforts

The analytical chemistry databases listed above include several examples of collections which have been put together in a single laboratory by systematically determining spectra of large numbers of chemical compounds for the specific purpose of building a database. The high quality collections of infrared spectra of compounds from the Aldrich and Sigma catalogues put together by Nicolet, an instrument manufacturer, are an example of this approach.

Another example is the integrated database system put together by the National Chemical Laboratory for Industry (Japan), which includes mass spectra, IR spectra, ^1^H and ^13^C NMR spectra, as well as ESR and Raman spectra, all determined in the NCLI laboratories under carefully controlled conditions [6]. In addition to providing an excellent example of a carefully constructed collection of spectra, this system also is perhaps the most fully realized example of a trend which will undoubtedly become important in the future—the use of integrated databases incorporating more than one kind of spectrum.

Databases put together under this strategy are generally of high quality, since the purity of the compounds used as well as the instrument parameters can be controlled by the party building the database. In the case of the integrated database, there is the further advantage that the correctness of the data can be cross-checked by examining complementary information obtained from different techniques.

In spite of the obvious advantages of this approach, however, it must be admitted that this type of database-building effort is expensive and relatively slow. The NCLI effort, for example, has required support for a laboratory effort including IR, mass spectral, and NMR instrumentation during approximately the past dozen years; the overall database index now contains 17,000 compounds [6]. The Thermodynamic Research Center at Texas A&M University sponsors a collection effort through laboratory measurements which generates about 75 spectra per year; again, the quality of the spectra is excellent, but one could never hope to build a large database by adding spectra at this rate.

### 3.2 Collections Put Together through Donations of Spectra from Diverse Laboratories

Many of the collections listed above have been put together by soliciting donations of spectra from many different laboratories. The Coblentz Society collection of IR spectra [7] and the American Petroleum Institute **(API)** Project 44 [la] collections of several kinds of spectra are examples of successful efforts of this nature. This approach has the obvious advantage that when a cooperative pool of donors exists, a database can be built relatively quickly and inexpensively.

On the other hand, when spectra are obtained from many different laboratories, there will inevitably be large variations in the quality of the data, not to mention differences in spectra due to the use of instruments of varying design. For example, the mass spectral collections include spectra from both magnetic sector and quadrupole instruments, which may have different types of mass discrimination, and therefore may give slightly different spectra for the same compound. However, the main problem associated with this collection technique is that completion of a collection project necessarily depends on the labor of volunteers. In general, the most successful efforts have been made when the management of a laboratory made the database collection a high priority work item (such as the petroleum industry’s generation of the API Project 44 Collection). When the effort is purely voluntary—something which is done only when other (high priority) work assignments have been completed—experience has demonstrated that the time-consuming task of preparing data for transfer to a collection is rarely actually undertaken.

### 3.3 Collection of Data from the Literature

A large number of scientific databases are composed by abstracting data from the scientific literature. This approach can also be applied to the construction of a spectral database for analytical use, thus obviating the need for achieving cooperation from donors of spectra. The most successful example of this type of database is the Wiley Registry of Mass Spectra, put together by F. W. McLafferty at Cornell University. As a result of the incorporation of spectra from the open literature, the database has grown dramatically in recent years, achieving as noted above a size of 123,704 spectra, up by about 50,000 over a period of some four or five years.

The database one obtains using this strategy, however, has a somewhat different nature from the databases built up through dedicated laboratory measurements or donations of spectra directly from the laboratories in which they were measured. The spectral data reported in scientific papers are often incomplete, either because the journals do not have sufficient space to publish entire spectra, or because the determination of a spectrum was not the primary motivation of the work reported in the literature. Therefore, a database built up with a large component of spectra from the scientific literature will include mainly partial spectra. The mean size of a mass spectrum in the Wiley Registry is 29 peaks, which can be compared with the mean size of the spectra in the NIST/EPA/MSDC Mass Spectral Database, 60 peaks (i.e., the mean size of the spectra taken from the literature is 13 peaks/spectrum).

## 4. Automated Evaluation of Spectral Collections

Spectral collections which are put together by laboratories which determine each individual spectrum are evaluated as they are built, and should not require much additional evaluation. However, when spectra come from a variety of sources, through donation schemes or literature acquisition, it is important to determine the quality of the spectra, and when a collection contains more than a few thousand spectra, it is obviously advantageous to have schemes whereby the spectral quality can be examined in some automated fashion. Such an approach to the evaluation of infrared spectra has recently been reported; a sheme was developed especially for use with the University of California-Riverside “Clearinghouse for Digital Infrared Spectra” [8]. Since this scheme is new, however, few details are available about its successes and/or failures when used with an actual database.

An automated evaluation scheme for mass spectra has been in use for many years, and the successful use of automated algorithms, as well as the kinds of problems which have been encountered, can be documented. The so-called Quality Index algorithm for mass spectra was originally proposed in 1978 by Speck, Venkataraghavan, and McLafferty [11], who put together an automated examination of various factors a trained mass spectrometrist would use in evaluating spectral quality. These included: (1) energy of the ionizing electrons; (2) presence of peaks at masses higher than the molecular weight of the compound; (3) presence of “illogical” peaks, which would not normally be formed in a compound of a particular formula; (4) whether or not relative isotopic abundances were correctly represented in the spectrum; (5) the total number of peaks in the spectrum (a measure of the completeness of the spectrum); (6) the mass of the lowest peak reported in the spectrum (another measure of completeness); and (7) the source of the spectrum.

Each factor was associated with a simple equation designed to give a numerical grade ranging from 0 to 1. For example, the so-called Quality Factor for the low mass limit was assigned by examining the mass of the lowest peak reported in the spectrum (*M*_min_) and comparing it to the molecular weight of the compound (*MW*), using the equation:

$$QF=(MW-M_{\min})/(MW-39)$$

(and QF was taken to be 1.0 for all compounds with molecular weight lower than 40). The final Quality Index (QI) for the spectrum was arrived at by multiplication:

$$QI=QF_{1}\cdot QF_{2}\cdot QF_{3}\cdot QF_{4}\cdot QF_{5}\cdot QF_{6}\cdot QF_{7}\cdot (1000).$$

Note that since the various factors are multiplied (rather than added) to achieve the final grade for a spectrum, a value of zero or a very low value for any single factor will lead to a low value for the spectrum as a whole. Furthermore, a spectrum receiving a rather high grade, but a grade less than unity, for each of the seven factors will end up with a low Quality Index value; (0.95)^7^ ×1000=698.

The same approach was used by scientists putting together the NIST/EPA/MSDC Mass Spectral Database [18,19], who omitted the seventh Quality Factor listed above (the source of the spectrum), and added some additional factors, namely; (1) stated sample purity; (2) whether or not the mass spectrometer has been calibrated for the measurement, and, if it has, the availability of the calibration data; (3) the presence of a peak at mass 28 (taken as evidence for the presence of air); (4) evidence for detector saturation; and (5) if the spectrum does not contain a peak having a mass equal to the molecular weight, the highest mass peak which is included (again, an indicator of the completeness of the spectrum).

In addition, many of the algorithms originally formulated by the Cornell team were modified for use with the NIH/EPA database. The modifications were based largely on analyses of the statistics of the individual quality factor values obtained for the spectra in the database. That is, it was assumed that (a) the standard deviation of the values obtained for any Quality Factor should be roughly proportional to the spectral significance of the property being measured; (b) the mean value of any given Quality Factor calculated for all the spectra in the database, should be 0.9 or greater, and (c) a Quality Factor should have a value of zero only in extreme cases. This is another way of saying that any Quality Factor which penalizes essentially all spectra in the database, or very few spectra, is not giving us any useful information for distinguishing between poor and good quality spectra. Thus, the modifications generally involved changing the equations to make the penalty greater or smaller, depending on the statistics observed. For example, the “low mass limit” Quality Factor given above was found to be weighted too strongly, and was modified to:

$$\begin{array}{l} QF=[(MW-M_{\min})/(MW-29){]}^{1/2}\text{for}\;MW<179,\\ \text{and}\;QF=[(MW+179-2M_{\min})/\\ (MW+179-58){]}^{1/2}\text{for}\;MW>179. \end{array}$$

In the NIST/EPA/MSDC database, until recently the protocol for putting together the data-base from the larger archive of spectra involved (1) calculating the Quality Index *(QI)* value for each spectrum in the system; (2) when there was more than one spectrum of a given compound, selecting from among those spectra by taking the one with the highest *QI* value for inclusion in the database. The spectra were not at any time examined visually by a mass spectrometrist; all judgements and selections were made using the automated procedure.

In general, the calculation is very effective in choosing between good spectra and poor spectra. However, in the 1986 edition of the database, it was noted that there were instances in which the algorithm led to the selection of a poor spectrum over several good spectra. In other cases, good spectra were found which had been assigned very low Quality Index values.

An analysis was made to identify the factors contributing to the observed problems. It was found, for instance, that spectra legitimately containing a large peak at *m/z* 28 were receiving low ratings because of the identification of that peak with the presence of air; the algorithm was modified to require the simultaneous presence of m/z 28 and *m/z* 32 with a ratio approximately the same as that one would observe for an air sample. Some of the fragmentation processes considered by the algorithm to be “illogical” were found to be important for certain types of compounds; as a result, all of the spectra of these compounds were receiving very low Quality Index values. For example, the “illogical loss” algorithm penalized all spectra in which there was an ion 2 mass-units below the parent molecular ion, that is, in which there was a fragmentation process consisting of a loss of H2 (or 2 H-atoms) from the molecular ion. This dissociation is very important for low molecular weight alkanes, and all alkane spectra were heavily penalized. The most abundant ion in the mass spectrum of ethane is at *m/z* 28 (C_2_H_4_^+^), and results from an “illogical loss” of two mass units, and therefore all spectra of ethane had Quality Index values of zero.

**Figure 1:**
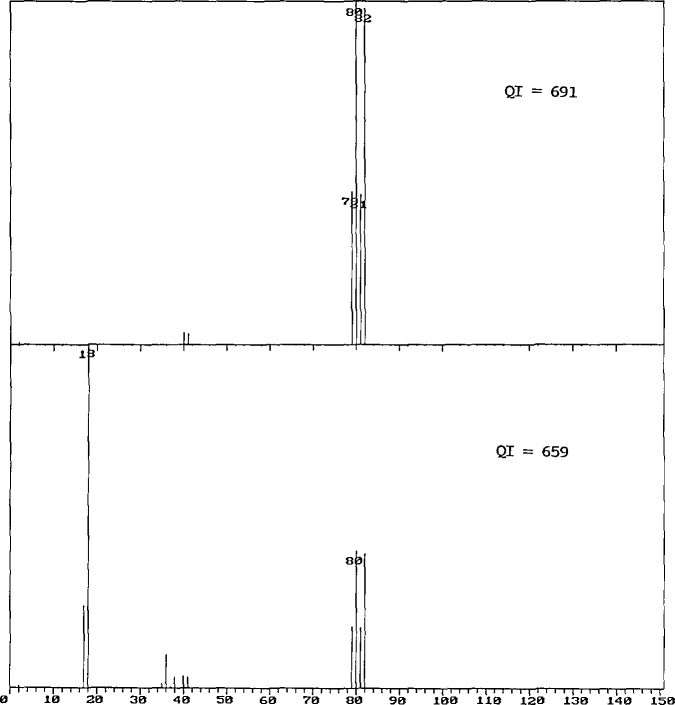
The mass spectrum of HBr shown on the bottom, containing water as the major component, was replaced by the spectrum shown on the top when the Quality Index calculation was revised (see discussion in text).

**Figure 2:**
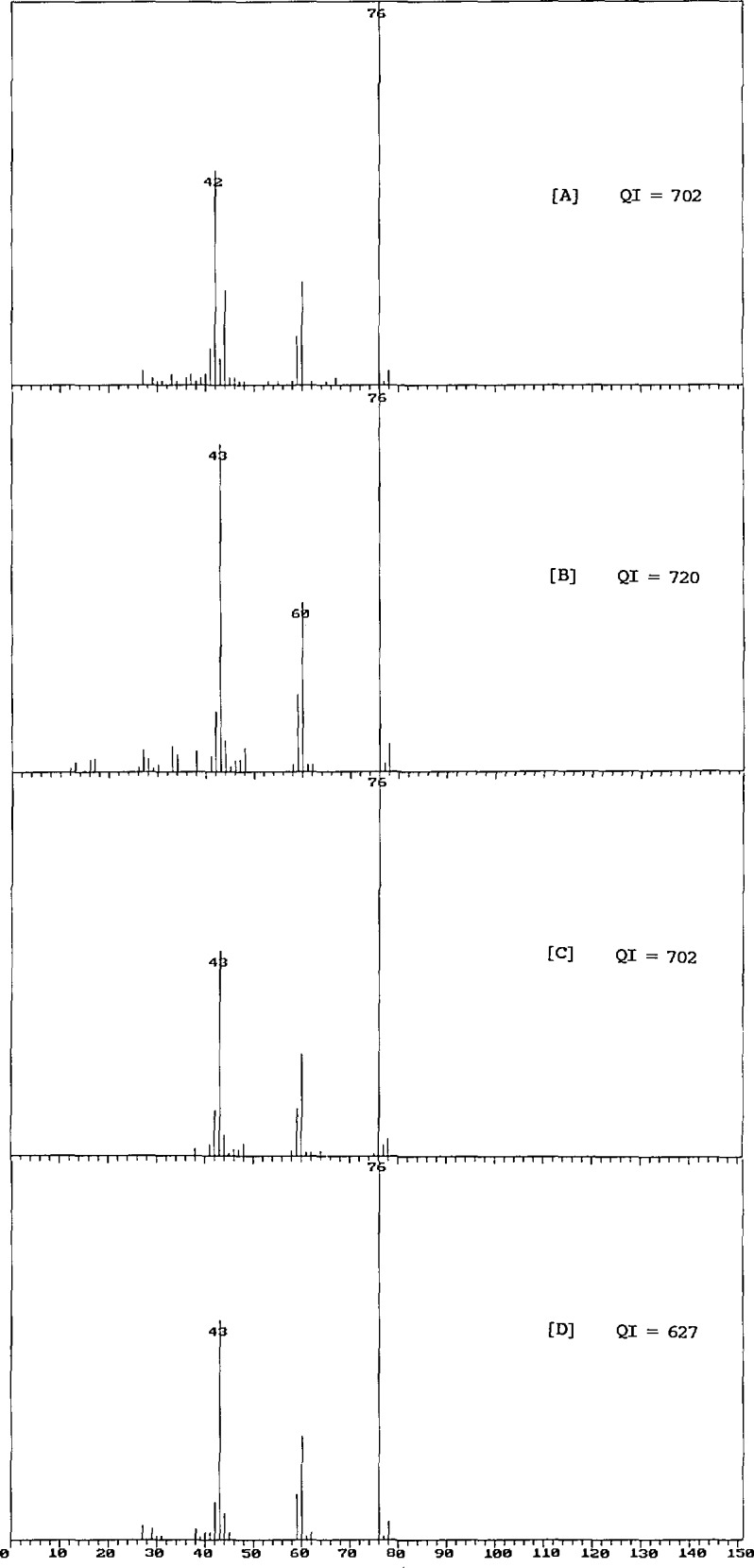
Four mass spectra of thiourea. In spectrum (A), *m/z* 43 has been misidentified as *m/z* 42; this is the spectrum originally selected by the *QI* calculation. Revision of the algorithm resulted in the choice of spectrum (B), which exhibits detector saturation. Spectra (C) and (D) (not selected by the program) are better quality spectra than (A) and (B).

**Figure 3:**
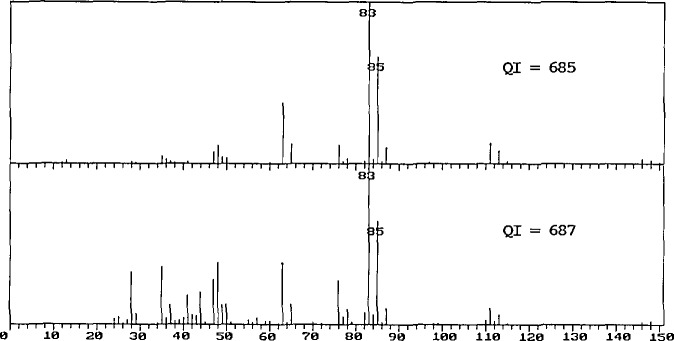
Two mass spectra of dichloroacetyl chloride exhibiting Quality Index values which differ by only 2 points. The lower spectrum, which has the higher *QI* value, contains peaks, for example at masses 41 and 44, which can only originate from an impurity.

Appropriate modifications to the algorithms were carried out, and the database was regenerated. The archive contains some 16,000 spectra which are replicates; the new calculation resulted in the replacement of 620 spectra by other spectra from the archive. A visual examination of these 620 pairs revealed that 50% of the changes had resulted in the selection of a spectrum of lower quality than that originally included in the database. Some of these replacement pairs are shown in figures 1–3.

Figure 1 shows two mass spectra of HBr. At the last revision of the Quality Index calculation, the spectrum on the top replaced the spectrum on the bottom which contains HCl impurity peaks and so much water that *m/z* 18 is the major peak. Note that although the current algorithm results in the choice of the better spectrum, the difference in the *QI* values between the good spectrum and the very bad spectrum is only 32 points

Figure 2 shows four spectra of thiourea. The spectrum on the top (A) is missing a major peak at *m/z* 43 (it appears that this peak has been misidentified as *m/z* 42), and an extra impurity peak at *m/z* 44 (or 45). That incorrect spectrum was formerly selected for the database; the “illogical fragmentation” algorithm did not recognize the incorrectly identified peaks. The spectrum (A) was replaced by the revised *QI* calculation with the spectrum (B) shown second, which now has a *QI* value 18 points higher than that of (A). Although spectrum (B) appears to be somewhat more complete than spectra (C) and (D), it clearly suffers from detector saturation, and therefore would be considered by an expert to be inferior in quality to both spectra (C) and (D). Curiously, the bad spectrum (A) receives the same *QI* grade as the good spectrum (C). Since the fragmentation of this parent ion does lead to the formation of an ion of *m/z* 42, it is unlikely that any algorithm could have detected the mistake in spectrum (A).

Figure 3 shows two spectra with Quality Index values which are within two points of one another. The spectrum with the higher *QI* value contains peaks, for example, at masses 41 and 44, which can only originate from an impurity.

An examination of these examples leads to the conclusion that *this type of Quality Index algorithm could not have done any better at selecting the best spectrum from among replicates.* With more fine tuning, this algorithm as it is presently constituted will never do any better. In setting up an evaluation-selection system of highly arbitrary equations, one is implicitly accepting that some statistical fraction of the spectra selected will be spectra which are not the best examples available in the archive. For instance, the recently-introduced Quality Factor, designed to penalize detector saturation, does so by searching for spectra having one or more additional peaks similar in magnitude to the base peak (peak of maximum abundance in a mass spectrum). Of course, some spectra legitimately have peaks of such magnitude, and they will be penalized; other spectra may be significantly saturated, but still pass such a test. The authors discuss this problem and conclude that *these errors can be tolerated* if the algorithm catches a large fraction of saturated spectra.

Until a truly “expert system” approach to the evaluation of analytical mass spectra is devised, it appears that the only possible procedure for selecting only the best available spectrum of each compound from an archive is to (1) use the existing Quality Index calculation as a rough first selection procedure, and (2) have an expert carry out a visual selection from among those replicate sets for which the Quality Index values are within 200–300 points of one another. This is the procedure now being carried out on the NIST/EPA/MSDC Mass Spectral Database, preparatory to release of the next update.
